# AMPK Alters Detrusor Contractility During Emptying in Normal Bladder and Hypertrophied Bladder with Partial Bladder Outlet Obstruction via CaMKKβ

**DOI:** 10.3390/ijms20112650

**Published:** 2019-05-29

**Authors:** Bo-Hwa Choi, Long-Hu Jin, Doo Yong Chung, Tae Jin Cho, Ju-Hee Kang, Tack Lee, Chang-Shin Park

**Affiliations:** 1 Department of Pharmacology, Hypoxia-Related Disease Research Center, Inha Research Institute for Medical Sciences, Inha University Colloge of Medicine, Incheon 22212, Korea; bo813@nate.com (B.-H.C.); johykang@inha.ac.kr (J.-H.K.); 2 Department of Urology, Inha University College of Medicine, Incheon 22332, Korea; jinlonghu_2021@hotmail.com (L.-H.J.); wjdendyd@gmail.com (D.Y.C.); 3 Department of Emergency Medicine, College of Medicine, Catholic Kwandong University, International St. Mary’s Hospital, Incheon 22711, Korea; kerygmata@hanmail.net

**Keywords:** AMPK, CaMKKβ, urinary bladder, muscle contraction, urethral obstruction

## Abstract

AMP-activated protein kinase (AMPK) has been implicated in contractility changes in bladders with partial bladder outlet obstruction (PBOO), but the role of AMPK in the contractile response of normal bladder remains unclear. We investigated the phosphorylation of AMPKα and expression of the involved upstream AMPK kinases (AMPKKs) in a model of bladders with PBOO and sought to determine whether the pharmacological inhibition of these two factors affected detrusor contractility in normal bladders, using female Sprague–Dawley rats. Cystometry and Western blot analysis were performed in rats that were subjected to PBOO induction or a sham operation. Cystometry was performed in normal rats that received selective inhibitors of AMPKα and Ca^2+^/calmodulin-dependent protein kinase kinase (CaMKKβ) (compound C and STO-609, respectively) at doses determined in the experiments. In the PBOO bladders, bladder weight and micturition pressure (MP) were higher and AMPKα phosphorylation (T172) and CaMKKβ expression was significantly reduced. Compound C and STO-609 increased MP. The increased contractile response in bladders with PBOO-induced hypertrophy was related to decreased CaMKKβ/AMPK signaling activity, and the pharmacological inhibition of this pathway in normal bladders increased detrusor contractility, implying a role of CaMKKβ/AMPK signaling in the bladder in the regulation of detrusor contractility.

## 1. Introduction

Muscle contractility refers to the ability of an organ to be shortened, which increases the pressure inside any organ that is wrapped with muscles three-dimensionally [1]. In the bladder, normal detrusor contractility during micturition is characterized by a continuous and sustained contraction that allows complete bladder emptying within an appropriate time span. In animals, after partial bladder outlet obstruction (PBOO), bladders show two types of altered contractility during micturition—compensated or decompensated bladder—with differences in the power, sustainability, and time span of contraction and the resultant residual urine volume [2,3]. Recent in vitro evidence obtained from animal models supports the hypothesis that regional tissue ischemia and decreased availability of energy for smooth muscle contraction (i.e., depletion of adenosine triphosphate [ATP]) play a significant role in post-PBOO changes in bladder contractility [4,5]. However, although multiple studies have been conducted on the magnitude, pattern, and time course of changes in bladder contractility after PBOO [2,4,6], it still remains unclear which factors play a key role in impaired contractility of the urinary bladder during micturition in bladders with PBOO. This issue is vitally important, because it is relevant to benign prostatic hyperplasia (BPH), a disease that affects many patients [7].

AMP-activated protein kinase (AMPK) is a cellular energy sensor that is activated in response to the depletion of ATP at the cellular and whole-body levels, as established in investigations of the heart [8,9]. AMPK is ubiquitously expressed intracellularly, similarly to other common protein kinases, and its activation requires phosphorylation by an upstream AMPK kinase (AMPKK) at the Thr-172 residue within the catalytic α subunit of AMPK [8,10]. To date, three mammalian upstream kinases for AMPK have been identified: liver kinase B1 (LKB1), AMPKK (described as a Ca^2+^/calmodulin–dependent protein kinase kinase, [CaMKKβ]), and TGF-β activated kinase-1 (TAK-1) [11]. Recent research has yielded new insights into the role of AMPK in controlling almost all cellular functions, including cell growth and proliferation, cell polarity, maintenance of mitochondrial homeostasis, and autophagy [12]. In particular, AMPK has been shown to play a functional role in controlling the contractility of the heart muscle [13], which led us to explore the possible role of AMPK in controlling the contractility of the bladder muscle.

The first evidence of the presence of AMPK in bladder tissue was obtained through cancer research [14], and its exact functional role in normal and diseased bladders has not been elucidated. In our previous study on bladders with PBOO, we showed for the first time that there is an inverse relationship between AMPK activity and detrusor contractility [15]. The phosphorylation of AMPK decreased in compensated bladders, which showed better contractility, and increased in decompensated bladders, which showed worse contractility. These findings raise the possibility that AMPK activity in the bladder may play a role in controlling detrusor contractility.

For the present study, our hypothesis was that AMPK activity in normal and diseased bladders directly or indirectly exerts effects on several downstream targets that regulate aspects of metabolism related to detrusor contractility, and the ability of normal bladder to change that activity after stress is related to changes in its compensated or decompensated condition. To investigate this hypothesis, we first investigated the activity of AMPK and AMPKKs by measuring the degree of phosphorylation and expression of these kinases in bladders with PBOO, compared to controls. Next, we determined whether the respective inhibitors of these two factors influenced the maximum bladder pressure related to micturition (MP) as an indicator of detrusor contractility during voiding in awake cystometry of normal rats. Finally, we compared the pre- and post-medication changes in cystometric parameters in response to continuous intravesical instillation with an inhibitor of AMPK or CaMKKβ in conscious normal rats.

## 2. Results

### 2.1. Structural Changes of Bladders with PBOO

**Figure 1:**
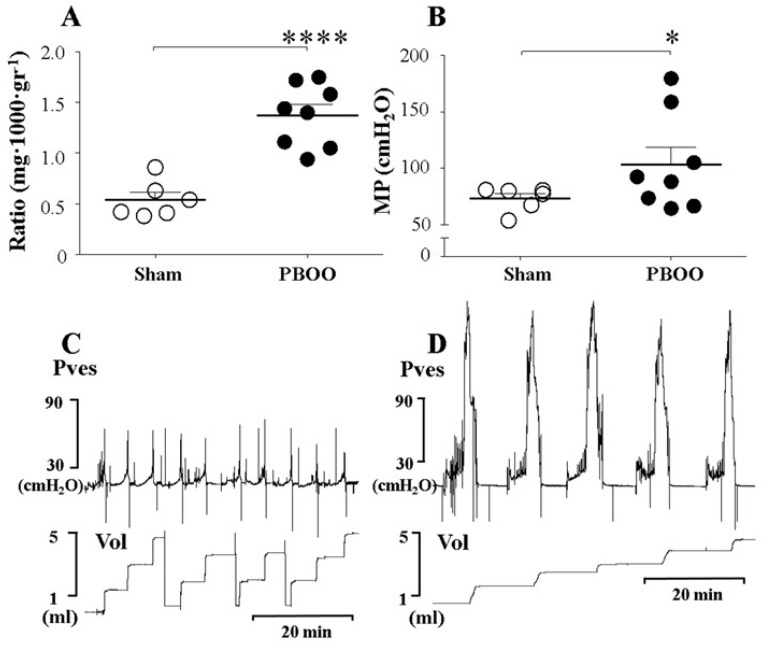
Comparison of bladder weight and micturition pressure (MP), with representative tracings, in sham rats and rats with partial bladder outlet obstruction (PBOO). (**A**). Ratio of bladder weight to body weight. (**B**). MP of sham rats and rats with PBOO. (**C**). Tracings of sham rats. (**D**). Tracings of rats with PBOO. The bladder weight and MP of rats with PBOO were significantly higher than those of sham rats. Representative tracings show the higher MP in rats with PBOO compared to sham rats. Pves: intravesical pressure. Vol: volume. *: *p* < 0.05 and ****: *p* < 0.0001, compared to sham.

PBOO maintained for two weeks resulted in a 2.5-fold increment in the bladder weight, manifested as the ratio between bladder and body weight, when compared to the sham-operated group (Table 1, Figure 1A). As shown previously, this increment is most likely to have resulted from hypertrophy and hyperplasia of the smooth muscle cells and connective tissues in the cross-sectioned bladder [16].

### 2.2. Functional Changes of Bladders with PBOO

The BaP, which represents the contractile response of the bladder during the filling phase, was higher in the PBOO group than in the sham group (Table 1). The MP, which represents the contractile response of the bladder during the voiding phase, was also higher in the PBOO group than in the sham group (108.8 ± 15.2 vs. 73.2 ± 4.4 cmH_2_O, *p* = 0.036) (Table 1, Figure 1B). This result suggests that the hypertrophied bladders that had experienced PBOO for two weeks showed pressure overload during both the filling and voiding phases. Seventy-five percent of the bladders (6 of 8) in rats with PBOO showed a residual urine percentage of less than 25%.

### 2.3. Immunoblot Characteristics of Bladders with PBOO

**Figure 2:**
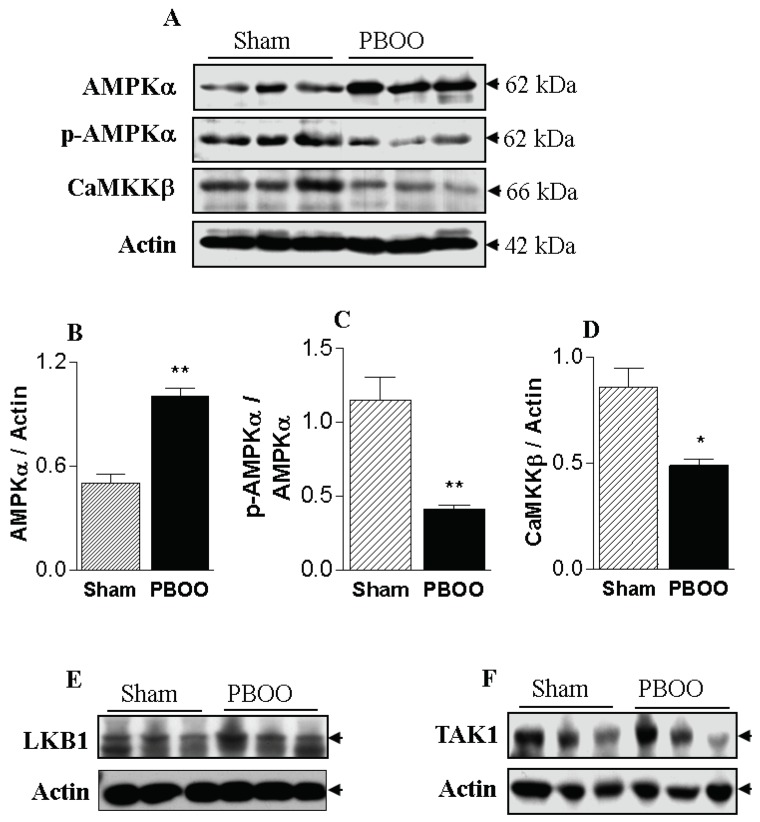
Effect of partial bladder outlet obstruction (PBOO) on AMPKα expression (**A**,**B**), AMPKα phosphorylation (**A**,**C**), CaMKKβ expression (**A**,**D**), and expression and phosphorylation of LKB1 (**E**) and TAK1 (**F**) in bladder tissue. The expression level of AMPKα increased significantly in response to PBOO, whereas the phosphorylation level, indicating AMPK activity, and CaMKKβ expression decreased significantly. *: *p* < 0.05 and **: *p* < 0.01, compared to sham.

The immunoblot analysis of the PBOO and sham-operated bladders showed significant differences in the level of expression and/or degree of phosphorylation of some signaling proteins (Figure 2). The protein expression levels of AMPKα in the bladders of PBOO rats were significantly higher than those of the sham group. In contrast to AMPKα expression levels, the degree of AMPKα phosphorylation in the bladders of PBOO rats was significantly lower than in the sham group. The increased expression and reduced phosphorylation of AMPKα imply that AMPK activity was reduced in the hypertrophied bladders with PBOO, which may be related to the increased voiding contractility of those bladders.

We examined three typical upstream kinases of AMPKα (LKB1, TAK1, and CaMKKβ) with the goal of identifying the changes in upstream kinase expression and regulation responsible for the reduced phosphorylation levels of AMPKα in response to PBOO. Changes in AMPK phosphorylation could not be connected with LKB1 and TAK1, because the expression of those kinases did not change. However, the protein expression levels of CaMKKβ were significantly lower in the bladders of the PBOO rats than in the sham group (Figure 2). Thus, the changes in AMPK phosphorylation are likely to be closely linked to changes in the expression of CaMKKβ, which is involved in the smooth muscle contraction pathway. These data indicate that the increase in the voiding contractile response of the PBOO bladders is related to decreased CaMKKβ/AMPK signaling activity.

### 2.4. In Vivo Investigation Using An Inhibitor of AMPK

**Figure 3:**
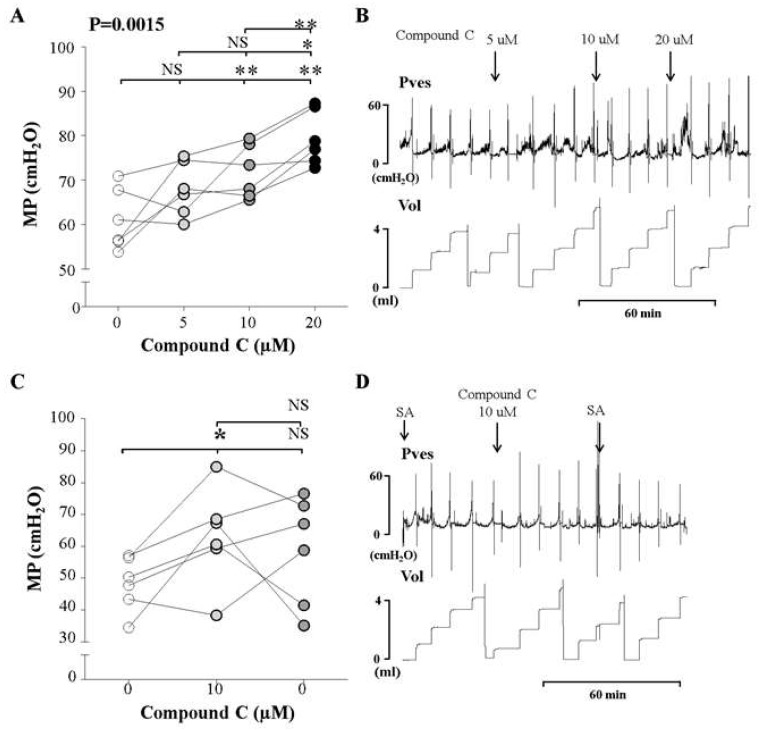
Effects and representative tracings of intravesical selective inhibitors of AMPK (compound C, A and B) with three consecutive increasing concentrations on the micturition pressure (MP) on cystometry, in order to identify the smallest dose showing a valid effect. NS: not significant, *p < 0.05 and **p < 0.01 compared to the control or the other dose; repeated one-way analysis of variance with the Dunnett post hoc test (**A**,**B**). Compound C increased the MP starting at an injected concentration of 10 μM, compared to the control group (**C**,**D**). The inhibitor was injected into the bladder, followed by normal saline. We did not use analysis of variance here, because this sub-experiment was conducted to compare the effects of injecting inhibitor and saline. Thus, we used the *t*-test for statistical comparisons between each event. NS: not significant, SA: saline. *: *p* < 0.05, compared to control (**C**).

We observed the effect of increasing intravesical concentrations of compound C (5, 10, and 20 μM) on the MP observed in awake cystometry of normal rats, in order to determine the lowest concentration that would increase the MP. The MP increased significantly after the injection of 10 and 20 μM compound C, compared to the findings of control cystometry, although no change was observed after administering 5 μM compound C. The MP in response to the 20 μM dose was higher than that observed after the 10 μM dose (Figure 3A,B). These findings suggest that the AMPKα inhibitor increased MP starting at a concentration of 10 μM, in a dose concentration-dependent manner.

After the determination of the effective dose, we performed cystometry and observed the effects of the determined dose (10 μM) of the inhibitor, followed by saline without the inhibitor. After the injection of 10 μM compound C, the MP increased compared to the control group. However, following saline instillation, the MP did not decrease (Figure 3C,D). This suggests that the voiding contractile response of the normal bladder increased as a result of AMPKα inhibition in the bladder.

### 2.5. In Vivo Investigation Using An Inhibitor of CaMKKβ, An Upstream Kinase of AMPK

**Figure 4:**
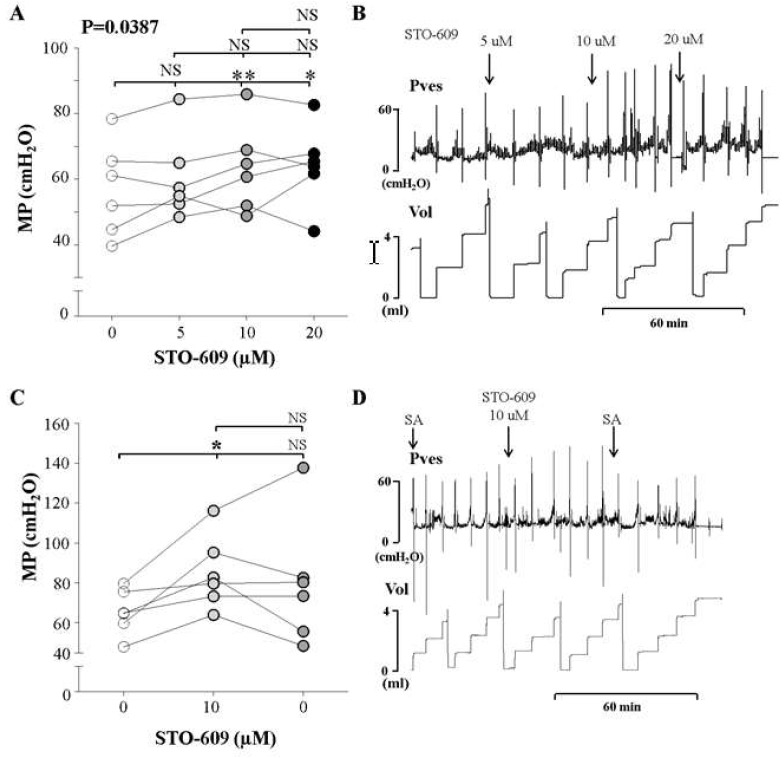
Effects and representative tracings of intravesical selective inhibitors of CaMKKβ (STO-609, **A**,**B**) with three consecutive increasing concentrations on the micturition pressure (MP) on cystometry, in order to identify the smallest dose showing a valid effect. NS: not significant, * *p* < 0.05 and ** *p* < 0.01 compared to the control or the other dose; repeated one-way analysis of variance with the Dunnett post hoc test (**A**). STO-609 increased the MP starting at an injected concentration of 10 μM, compared to the control group (**C**,**D**). The inhibitor was injected into the bladder, followed by normal saline. We did not use analysis of variance here, because this sub-experiment was conducted to compare the effects of injecting inhibitor and saline. Thus, we used the *t*-test for statistical comparisons between each event. NS: not significant, SA: saline. *: *p* < 0.05, compared to control (**C**).

We observed the effects of increasing concentrations of intravesical STO-609 (5, 10, and 20 μM) an inhibitor of CaMKKβ, on the MP of awake normal rats using cystometry. The MP increased significantly after the injection of 10 μM STO-609 compared to the findings of control cystometry, although there was no significant change after the injection of 5 and 20 μM STO-609 (Figure 4A,B). These findings suggest that the CaMKKβ inhibitor increased the MP at a concentration of 10 μM.

Additionally, we compared the effects of the determined dose (10 μM) of the inhibitor and saline without the inhibitor. After injection of 10 μM STO-66, the MP increased compared to the control group. However, following saline instillation, the MP did not decrease (Figure 4C,D). This suggests that the voiding contractile response of the normal bladder increased in response to CaMKKβ inhibition in the bladder.

## 3. Discussion

Our study demonstrated that hypertrophied bladders with PBOO showed a stronger contractile response during the emptying phase, as well as lower AMPKα activity, as indicated by the reduced phosphorylation of AMPKα. Among the three upstream AMPKKs, only CaMKKβ showed reduced expression. This implies that reduced CaMKKβ/AMPKα activity in the bladder may be responsible for the increased bladder contractility during emptying observed after stresses such as PBOO. Based on these results, we applied intravesical inhibitors (Compound C and STO-609) to awake normal rats, and confirmed that they had the effect of increasing MP, which served as an indicator of detrusor contractility during emptying. Thus, AMPKα seems to play a significant functional role in the control of detrusor contractility during emptying in normal rats, as well as in rats with PBOO.

BPH is a common progressive urological condition for elderly men, occurring in up to 90% of men by the age of 80 [7]. PBOO, caused by this aging process, may lead to alterations of bladder contractility, such as overactive bladder [17] and underactive bladder [18]. The bladder contractility changes observed in men with BPH can be reproduced in animal models by a surgical obstruction with a ligature around the urethra [3,6]. In this model, surgical obstruction results in a rapid increase in bladder weight and the ability to generate higher pressure to overcome the obstruction, which was confirmed through our experimental results. After approximately 14 days, bladder function is stabilized through compensatory changes, and then decompensation occurs, resulting a loss of the ability to empty the residual urine completely [2]. However, our previous study [6] showed that decompensatory changes, as defined by the residual urine volume, can occur at an earlier phase, such as 1 week after PBOO. Furthermore, a tendency was found for the frequency of bladder decompensation to decrease from 62.5% at one week after PBOO induction to 33.3% at two weeks. This led to the hypothesis that the decompensatory response does not always follow the compensatory response, and that the determination of compensation or decompensation depends on individual cellular characteristics relevant for adaptation to the conditions of PBOO. This raises the possibility that the individual response of the normal bladder in terms of AMPK inhibition after stressors such as PBOO may determine whether compensation or decompensation occurs. Thus, it is of vital importance to determine the critical molecular determinant among the various cellular signaling pathways that could be involved in the outcomes of bladders with PBOO, although the restricted and fragmentary evidence does not always allow us to clearly interpret relevant findings.

The urinary bladder, composed of a musculomembranous wall, needs an adequate and continuous energy supply to its smooth muscle cells in order to maintain normal contractility function. Their primary energy source, ATP, is mainly generated through oxidative phosphorylation of mitochondria in aerobic conditions and glycolysis in anaerobic conditions [19]. Although the coiled shape of the bladder vessel is known to allow it to stretch in response to being filled with urine without a significant decrease in peripheral perfusion, the bladder can experience periodic ischemia even in normal conditions when intravesical or intraabdominal pressure increases above the vascular capillary pressure [20]. However, the intracellular ATP content in several types of smooth muscle was found to be maintained at a constant level during a single isometric contraction [21,22]. In order to keep ATP at a constant level, the ATP consumed during the previous contraction must be restored through rapid synthesis. The mechanism underlying this maintenance is not yet fully understood, but it seems that a dynamic feedback mechanism in the bladder must be necessary to monitor and rapidly control intracellular energy levels. AMPK is a logical candidate for this role, and it is best known for its role as a key sensor of intracellular energy in almost all eukaryotes [8].

PBOO results in both morphological and bioenergetic changes in the normal bladder as it adapts to a new environment [2,3,5]. Morphologically, the obstructed bladder undergoes smooth muscle hypertrophy and remodeling, shown in our results by a 2.5-fold increase of bladder weight. At the molecular level, the hypertrophied bladder is characterized by an increase in gene transcription and translation in smooth muscle, suggesting increased protein synthesis [2]. Bioenergetically, investigators reported that bladders with PBOO showed a significant decrease in the tissue content of ATP and an increased level of AMP [5]. This is the prerequisite condition for the activation of AMPK. Thus, we predicted that we would observe increased activity of AMPK in hypertrophied bladders that had experienced two weeks of obstruction, but instead, we observed the inverse pattern (reduced activity). This suggests that a distinct or additional mechanism may be involved in the interplay between AMPK activity and the control of protein synthesis or contractility during voiding. Further research into the acknowledged gaps in this area is needed in the future.

AMPK acts as a metabolic regulator that is activated by cellular stresses, causing energy deprivation [8,23]. This condition, reflected by an increase in the AMP:ATP ratio, leads to conformational changes in AMPK that make it more susceptible to phosphorylation and activation by an AMPK kinase [8]. Once activated, AMPK directly inhibits other biosynthetic and related pathways that consume ATP, but are not acutely required for survival, although it also promotes catabolic pathways that enhance ATP production. Thus, AMPK has been known to play a prominent role both as an energy sensor and as a multifunctional metabolic regulator [23]. Given that protein synthesis is a high energy-consuming process, Chan et al. showed that pharmacological activation of AMPK inhibits protein synthesis associated with cardiac hypertrophy [24]; these findings can be applied to the bladder, but in the opposite direction. Our results demonstrated that the significant reduction of AMPK activity shown in Figure 2 was accompanied by bladder hypertrophy, representing increased protein synthesis. The PBOO-derived mechanical stretch stress caused by increased tension during voiding triggers the bladder hypertrophy through modification of gene expression and transcription in bladder cells and through deregulation of the synthesis of many proteins and enzymes. AMPK seems to have a counteracting action against these effects of PBOO by inhibiting the protein synthesis in hypertrophied bladder.

AMPK is also known to be relevant as a latent regulator of vascular smooth muscle tone, although this is not precisely representative of contractility [25]. In a study using vascular smooth muscle cells, its activation by metformin attenuated the phenylephrine-induced contraction of smooth muscle. This study found that AMPK played an intermediary role in regulating the maximal contraction level, similar to the brake pedal of a running car. Although it has not been determined whether AMPK plays a direct inhibitory role in the regulation of metabolic pathways underlying the contraction of bladder smooth muscle during voiding, our study provides indirect evidence supporting a view of AMPK as a regulator of detrusor contraction during the emptying phase. In our results, the pharmacological inhibition of AMPK and CAMKKβ increased the voiding contractile response of the normal bladder in awake rats, and after PBOO, compensated bladders showed decreased AMPK and CaMKKβ activity. These suggest that AMPK attenuates voiding contraction, although the intermediate process is unknown.

A limitation of this study is the lack of anatomic evidence concerning this role of AMPK in the bladder. Despite this lack of solid evidence, trends in some studies support our result. Koeck et al. [26] suggested that the heart and the urinary bladder are similar hollow muscular organs, and that similar mechanisms of pressure overload injury in pathological conditions such as hypertension and PBOO can lead to organ failure via induced hypertrophy, fibrosis, and loss of contractility. Additionally, they concluded that some biochemical findings in the heart provide a valuable source of information for investigators working on the bladder. Furthermore, Zhang et al. [27] reported that AMPK activation could regulate the expression of connexin 43 (Cx43), one of the GAP junctions, and suggested that the overexpression of Cx43 in their cyclophosphamide-induced interstitial cystitis model could be regulated by AMPK activation, controlling overactive bladder dysfunction. Another limitation of our study is the relatively small number of obstructed rats and the narrow spectrum of disease severity. The obstructed bladders in our study included more compensated bladders, with higher pressure and less residual urine (75%), than other studies [14,20] Because of the dynamic nature of the bladder response after PBOO, variability in obstruction severity and host response, and the complexity of interactions between the bladder and urethra, this kind of study could be limited to isolated ‘snapshots’ in time during the disease process. Furthermore, our sample size was not large enough to reach definitive conclusions about this subject. Despite these limitations, this study provides information on the general spectrum of AMPK-targeting strategies that could be developed as novel therapeutic approaches for treating bladder dysfunction.

## 4. Materials and Methods

### 4.1. Experimental Animals and Surgical Procedures

Thirty-eight female Sprague–Dawley rats weighing between 180 and 200 g obtained from Orient Bio Inc. (Gyeonggi-do, Korea) were used in this study.

Anesthesia was induced by ketamine (ketamine; Yuhan Corp, Seoul, Korea; 75 mg/kg intraperitoneally) and xylazine (Rompun; Bayer Korea Corp, Korea; 15 mg/kg intraperitoneally). The PBOO rat model was prepared following a previously described procedure [16]. The catheterization methods are described in our previous work [28]. Briefly, a polyethylene catheter (PE-50; Becton Dickinson, Parsipanny, NJ, USA) with a cuff was implanted into the dome of the bladder, and tied in place with a purse-string suture. The catheter was tunneled subcutaneously and anchored to the skin of the back of the neck with a silk ligature. The free end of the catheter was sealed.

Three separate sets of experimental trials were performed. In the first main experiment, 14 rats underwent PBOO induction (*n* = 8) or a sham operation (*n* = 6). We observed the functional differences between those two groups of bladders using cystometry. Immediately afterward, the bladders were removed and analyzed using immunoblotting. The protein expression and phosphorylation level of AMPKα and expression of three typical upstream kinases of AMPKα (LKB1, TAK1, and CaMKKβ) were investigated using western blot analysis.

Secondly, functional changes of the normal bladder were observed in response to the intravesical instillation of reagents that can mimic the conditions of bladders with PBOO. Cystometry was performed with selective inhibitors (Compound C and STO-609) for AMPKα (*n* = 6) and CaMKKβ (*n* = 6). Because did not know which doses of the reagents were valid, we used three consecutive increasing concentrations.

In the third set of experiments, cystometry was performed in 12 normal rats (AMPKα [*n* = 6] and CaMKKβ [*n* = 6]) using the concentration of inhibitors that was determined to be most appropriate in the second set of experiments.

The rats were maintained under standard conditions, with a 12:12-h light:dark cycle and free access to tap water and food pellets. The animals were placed individually in cages and maintained in the same manner after surgery. All procedures were conducted in accordance with the Guide for the Care and Use of Laboratory Animals of the National Institutes of Health, and the experimental protocol was approved by the Animal Ethics Committee of the Inha University College of Medicine. (INHA 161017-448)

### 4.2. Reagents

AMPKα, p-AMPKα, LKB1, and TAK1 antibodies were purchased from Cell Signaling Technology (Beverly, MA, USA), and CaMKKβ from Santa Cruz Biotechnology (Santa Cruz, CA, USA). Beta-actin antibody was purchased from Sigma-Aldrich (St. Louis, MO, USA).

The AMPK inhibitor compound C and CaMKKβ inhibitor STO-609 were purchased from Calbiochem (Merck KGaA, Darmastadt, Germany) and Sigma-Aldrich, respectively.

### 4.3. Cystometric Investigations

Cystometry was performed without any anesthesia three days after catheter implantation. Conscious rats were placed in metabolic cages without restraint, and a bladder catheter was connected via a T-tube to a pressure transducer (Research Grade Blood Pressure Transducer, Harvard Apparatus, Holliston, MA, USA) and a microinjection pump (PHD22/2000 pump; Harvard Apparatus). Volumes related to voiding were recorded with a fluid collector connected to a force-displacement transducer (Research Grade Isometric Transducer, Harvard Apparatus). Room-temperature saline was infused into the bladder continuously at a rate of 20 mL/h in rats with PBOO or at a rate of 10 mL/h in normal rats. The pressure and micturition volumes were recorded continuously with Acq Knowledge 3.8.1 software and an MP150 data acquisition system (BIOPAC Systems, Goleta, CA, USA) at a sampling rate of 100 Hz. The average values from three reproducible micturition cycles were used for the analysis. The following urodynamic pressure- and volume-related parameters were investigated: basal pressure (BaP: the lowest bladder pressure during filling), threshold pressure (TP: bladder pressure immediately before micturition), MP, micturition volume (MV: volume of expelled urine), residual volume (RV: bladder capacity minus MV), bladder capacity (BC: MV + RV), and micturition interval (MI).

### 4.4. Immunoblot Analysis

The procedures for the immunoblot analysis were performed as described previously (15). Bladder tissues were homogenized in liquid nitrogen and sonicated in 30% (*w/v*) lysis buffer (320 mM sucrose, 200 mM HEPES, and 1 mM EDTA) containing a phosphatase inhibitor cocktail (PhosSTOP, Roche, Germany) and a protease inhibitor cocktail (Sigma-Aldrich). The protein concentration of the crude supernatants was determined with the Pierce BCA Protein Assay kit (Pierce, Rockford, IL, USA).

### 4.5. Statistical Analysis

The results are given as mean values ± standard errors of the mean (SEM). The normality of the distribution was confirmed using the Shapiro–Wilk W test. Statistical significance was determined using the unpaired or paired Student *t*-test or one-way analysis of variance (ANOVA) with the Tukey post-hoc test for multiple comparisons. Statistical analyses were performed with GraphPad Prism (version 7; GraphPad Prism Software. Inc., San Diego, CA, USA), and *p-*values <0.05 were considered to indicate statistical significance.

## 5. Conclusions

In summary, the present study demonstrated that the increased contractile response in bladders with PBOO was related to decreased CaMKKβ/AMPKα signaling activity. Furthermore, the inhibition of AMPKα and CaMKKβ, respectively, increased the MP, an indicator of increased detrusor contractility during normal voiding. Our results provide a rationale for targeting this CaMKKβ/AMPKα pathway to control detrusor contractility, and suggest that this approach may be promising for restoring bladder contractility.

## Figures and Tables

**Table 1:** Comparison of cystometric parameters between the sham rats and rats with partial bladder outlet obstruction (PBOO).

	Sham (*n* = 6)	PBOO (*n* = 8)
Ratio (mg·1000·gr^−1^)	0.54 ± 0.08	1.37 ± 0.11 ***
BaP (cmH_2_O)	12.07 ± 0.55	15.90 ± 1.61 *
TP (cmH_2_O)	29.47 ± 3.23	46.06 ± 6.41
MP (cmH_2_O)	73.18 ± 4.43	108.8 ± 15.16 *
BC (mL)	1.16 ± 0.13	2.57 ± 0.36 **
MV (mL)	1.15 ± 0.13	2.09 ± 0.34 *
RV (mL)	0.01 ± 0.01	0.49 ± 0.28
MI (min)	3.59 ± 0.42	7.70 ± 0.93 **

Ratio: ratio of bladder weight to body weight, BaP: basal pressure, TP: threshold pressure, MP: micturition pressure, BC: bladder capacity, MV: micturition volume, RV: residual volume, MI: micturition interval. *: *p *< 0.05, **: *p *< 0.01, ***: *p *< 0.001, compared to sham.

## Abbreviations

AMPK	AMPK-activated protein kinase
PBOO	Partial bladder outlet obstruction
AMPKKs	AMPK kinases
CaMKKβ	Ca^2+^/calmodulin-dependent protein kinase kinase
LKB1	Liver kinase B1
TAK1	TGFβ-Activated Kinase 1
ATP	Adenosine triphosphate
BPH	Benign prostatic hyperplasia
MP	Maximum bladder pressure related to the micturition
BaP	Basal pressure
TP	Threshold pressure
MV	Micturition volume
RV	Residual volume
BC	Bladder capacity
MI	Micturition interval
